# Clinical features and outcomes of patients with follicular lymphoma: A real-world study of 926 patients in China

**DOI:** 10.3389/fonc.2022.863021

**Published:** 2022-09-16

**Authors:** Fenghua Gao, Tingting Zhang, Xia Liu, Zhenjie Qu, Xianming Liu, Lanfang Li, Lihua Qiu, Zhengzi Qian, Shiyong Zhou, Wenchen Gong, Bin Meng, Xiubao Ren, Xianhuo Wang, Huilai Zhang

**Affiliations:** ^1^ Department of Lymphoma, Tianjin Medical University Cancer Institute and Hospital, National Clinical Research Center of Cancer, Key Laboratory of Cancer Prevention and Therapy, Tianjin’s Clinical Research Center for Cancer, Sino-US Center for Lymphoma and Leukemia Research, Tianjin, China; ^2^ Department of Pathology, Tianjin Medical University Cancer Institute and Hospital, Tianjin, China; ^3^ Department of Immunology/Biotherapy, Tianjin Medical University Cancer Institute and Hospital, Tianjin, China

**Keywords:** follicular lymphoma, real-world, Chinese, POD24, histological transformation

## Abstract

**Background:**

The data about the clinical features and outcomes of Chinese patients with follicular lymphoma (FL) are limited. Here, we conducted a retrospective study to explore the initial treatment strategies and clinical outcomes of Chinese patients with FL in the real world.

**Method:**

This study included FL patients who were newly diagnosed in Tianjin Medical University Cancer Institute and Hospital from March 2002 to August 2020.

**Results:**

A total of 926 FL patients were enrolled. The median age was 54 years old, and the majority of the Chinese FL patients had advanced-stage disease and Eastern Cooperative Oncology Group(ECOG) <1 but less frequently infiltrated bone marrow. After a median of 38-month follow-up, the 5-year progressive-free survival (PFS) and overall survival (OS) of grade1–3a were 57.8% and 88.7%, respectively, which both are similar to those reported in previous Chinese and Western studies. The co-existence at diagnosis of FL and diffuse large B-cell lymphoma (DLBCL) components (FL/DLBCL) was associated with poor outcomes. The FL grades and proportion of DLBCL component in FL/DLBCL did not have an impact on PFS and OS. The most common regimen with great efficacy and risk–benefit was RCHOP-like followed by R maintenance regimen. The 5-year cumulative hazard of histological transformation (HT) was 4.7% (95% CI, 3.5–5.9); median time to transformation was 23.5 months (range, 2–146 months) after diagnosis. Three-year survival following transformation was 55% (95% CI, 40–70). Patients with stage III–IV, elevated β2 microglobulin (β2-MG), and B symptoms seemed to be more prone to progress within 24 months of frontline therapy (POD24). The FLIPI-2 showed the highest specificity to predict POD24, reflecting the prediction of correctly classifying as low-risk patients, but the FLIPI had the highest sensitivity to predict the risk of progression for critical patients.

**Conclusions:**

We revealed the clinical characteristics and outcomes of FL patients in the real world in China, which may provide novel data on prognostic factors and primary treatment of FL, applicable to routine clinical practice.

## Introduction

Follicular lymphoma (FL) is a malignant tumor that originates from B cells in the center of the follicle, and it is characterized by heterogeneous clinical evolution (1). It is the second most common non-Hodgkin lymphoma (NHL) subtype in the USA with more than 14,000 cases diagnosed annually (2), accounting for 20%–25% of all lymphomas (3). FL is clinically indolent; most patients are sensitive to chemotherapy or immunochemotherapy (IC), but even in the era of rituximab, FL continues to be considered incurable despite the improvements in overall survival (OS) observed over the past few decades, and the clinical presentations and outcomes between individuals are still heterogeneous (4). For patients with symptomatic diseases, such as bulky disease (one lymphoma lesion >7 cm); three separate nodes of 3 cm or more; symptomatic splenic enlargement; organ compression by tumor, pleural, or peritoneal effusion; raised serum concentrations of either lactate dehydrogenase or β2-microglobulin; or the presence of B symptoms, need standard of care (5). Immunochemotherapeutic regimens are the most effective treatment of FL in the first-line setting (6). In patients who are asymptomatic, treatment options are still under debate (7).

Transformation of FL (tFL) to a more aggressive histology, usually into diffuse large B-cell lymphoma (DLBCL), is an important clinical event associated with poor outcomes (8, 9). The annual risk of histological transformation (HT) has been estimated to be 2%–3% per year. The identification of a significant DLBCL component at FL diagnosis is a common occurrence. The co-existence of both FL and DLBCL components (FL/DLBCL) in the same biopsy has been considered as “transformed lymphoma at diagnosis,” thus leading to the diagnosis of histological transformation (10). Some scholars directly conclude that this is an “early transformation” (11). Others consider that, in some cases, an indolent and aggressive component co-exist at diagnosis, a condition often referred to as “composite” lymphoma if the different histologies are detected within a single lesion (12).

Scholars in Western countries have made a detailed description of the demographics, baseline disease characteristics, initial treatment strategies, and results of patients with FL (13, 14). As far as this part is concerned, data on Chinese patients with FL are limited. Here, we conducted a retrospective study to explore the initial treatment strategies and clinical outcomes of Chinese patients with FL in the real world.

## Patients and methods

### Patients

A total of 1,006 patients were retrospectively screened and reviewed by two experienced hematopathologists for diagnostic confirmation at Tianjin Medical University Cancer Institute and Hospital (TMUCIH) between March 2002 to August 2020, and they all met the WHO classification criteria for lymphoid tissue tumors. All cell types (small-cell, mixed, or large-cell FL) could be included in the study. We excluded patients who were <18 years old, patients who lacked adequate clinical information, or those who were lost to follow-up. The clinical data are complete. Staging procedures included at least CT scans of the chest and whole abdomen, or superficial lymph node ultrasound, bone marrow biopsy, routine blood counts, and biochemical examinations. The study was approved by the institutional review boards.

### Data collection

Data collection includes detailed demographic characteristics, initial staging, and clinical and biological characteristics. Patients were staged according to the Lugano classification criteria (15). Bone marrow biopsy (BMB) has been the standard in lymphoma staging, so unilateral BMB was performed, along with bone marrow smear and flow cytometry in all eligible FL patients. FL/DLBCL is defined as the presence of variable DLBCL components in lymph node biopsies of other patients diagnosed with FL. According to WHO recommendations, the DLBCL component was defined as an area of large cells in sheets lacking follicular architecture assessed by staining for follicular dendritic cells (CD21 or CD23) (16). The Hans algorithm was used for COO assignment by IHC (17). The patient’s FLIPI, FLIPI 2, and PRIMA-PI were calculated based on baseline characteristics.

Initial treatment was defined according to the intention to treat principle. All patients meet at least one treatment indication in the FL study group (specific treatment indications are shown in **Supplementary Table S1**). Watch and wait (WW) was defined as the decision not to treat patients and also by the absence of treatment within the first 3 months of follow-up. Transformation was defined on the basis solely of pathological criteria. At the time of clinical relapse or progression or clinical suspicion of tFL, a lymph node biopsy was made whenever feasible. Time to histological transformation was calculated from the date of FL diagnosis to the date of HT. Survival following transformation (SFT) was calculated as the time of HT documentation until death from any cause or last follow-up. POD24 was broadly defined as progression of disease within 24 months of first-line therapy (18).

### Statistical analysis

Statistical analyses were performed in SPSS version 22 (SPSS, Inc., Chicago, IL, USA). Demographics, baseline disease characteristics, and initial treatment strategy for FL patients in the entire cohort were summarized using descriptive statistics (medians and ranges for continuous variables; frequencies for categorical variables). Associations between POD24 and clinical factors were evaluated using the χ^2^ test.

OS was defined as the time from initial diagnosis until death from any cause, and progression-free survival (PFS) was defined as the time from diagnosis to disease recurrence, disease progression, or death. Kaplan–Meier method was used for the analysis of survival, and differences were assessed using the log-rank test. A two-tailed p<0.05 was considered statistically significant.

## Results

### Patients and disease characteristics

**Figure 1:**
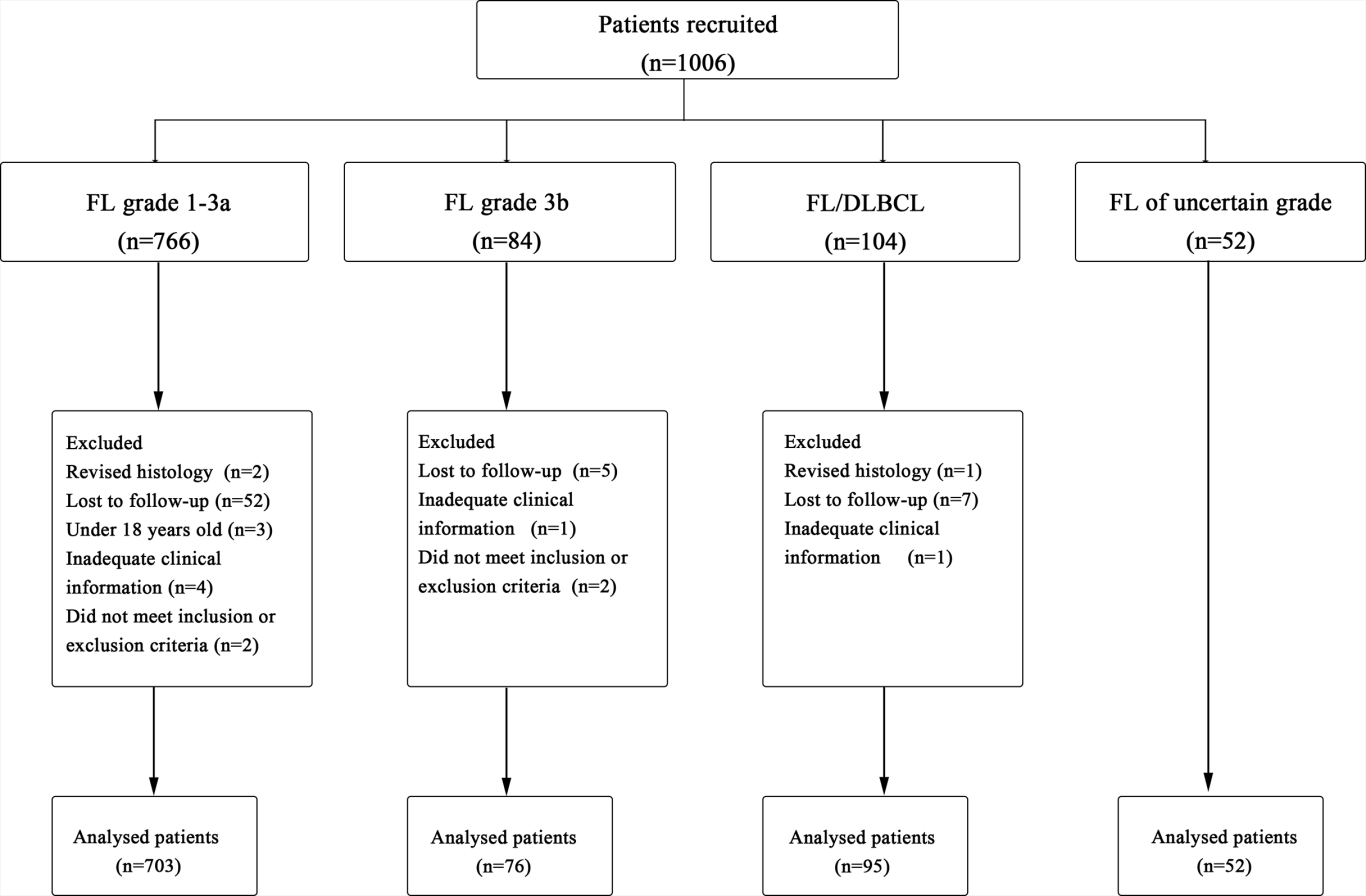
Procedure of study cohort selection and number of patients included in analysis. FL, follicular lymphoma; DLBCL, diffuse large B-cell lymphoma.

A total of 1,006 cases were originally identified; 80 patients were excluded for not meeting the criteria resulting in 926 patients for analysis (**Figure 1**). Baseline characteristics of 926 FL patients are presented in **Table 1**. Median age was 54 (range, 18–90) years with 33.7% of patients aged ≥60 years, and 47.2% were male; most of them had disseminated stage III–IV disease (69.4%). A total of 90.9% (n=842) of patients had ECOG ≤ 1, but 1.8% (n=17) presented with decreased functional status with ECOG 2–4.

**Table 1:** Baseline characteristics of the patients with FL (N=926).

Characteristic	n (%)	
Age (year), median (range)	54 (24-90)	
<60	613 (66.3%)	
≥60	313(33.7%)	
Gender		
Female	492 (52.8%)	
Male	434 (47.2%)	
Disease stage		
I/II	183 (19.8%)	
III/IV	643 (69.4%)	
Missing	100 (10.8%)	
Histological grade		
1–3a	703 (75.9%)	
3b	76 (8.2%)	
FL/DLBCL	95 (10.3%)	
FL of uncertain grade	52 (5.6%)	
ECOG		
0–1	842 (90.9%)	
2–4	17 (1.8%)	
Missing	67 (7.3%)	
B symptoms		
Yes	128 (13.8%)	
No	730 (78.8%)	
Missing	68 (7.4%)	
Bone marrow involvement		
Yes	108 (11.7%)	
No	809 (87.4%)	
Missing	9 (0.9%)	
Bulky disease (cm) >6cm		
Yes	699 (75.5%)	
No	144 (15.6%)	
Missing	83 (8.9%)	
Extranodal involvement ≥1		
Yes	327 (35.3%)	
No	503 (54.3%)	
Missing	96 (10.4%)	
β2-MG (ng/L)>UNL		
Yes	275 (29.7%)	
No	648 (70.0%)	
Missing	3 (0.3%)	
LDH(u/L)>UNL		
Yes	207 (22.4%)	
No	719 (77.6%)	
HB(g/L) <120		
Yes	177 (19.1%)	
No	743 (80.2%)	
Missing	6 (0.7%)	
FLIPI		
0–1	234 (25.3%)	
2	283 (30.6%)	
3–5	305 (32.9%)	
Missing	104 (11.2%)	
FLIPI-2		
0	305 (32.9%)	
1–2	456 (49.2%)	
3–5	109 (11.8%)	
Missing	56 (6.1%)	
PRIMPI		
Low risk	616 (66.5%)	
Intermediate risk	50 (5.4%)	
High risk	229 (24.7%)	
Missing	31(3.4%)	

FL, follicular lymphoma; DLBCL, diffuse large B-cell lymphoma; ECOG, Eastern Cooperative Oncology Group; β2-MG, β2 microglobulin; LDH, serum lactate dehydrogenase; Hb, hemoglobin; FLIPI, FL International Prognostic Index.

FL involved the bone marrow in 11.7% (n=108) of patients, and 13.8% (n=128) of patients had evidence of B symptoms at diagnosis, including fevers, weight loss, and night sweats. Additionally, 13.6% (n=126) of FL patients had extranodal disease involvement ≥1. For laboratory data, 22.4% (n=207) patients had elevated lactate dehydrogenase (LDH), 19.1% (n=177) had hemoglobin (Hb) <120 g/L, and 29.7% (n=275) patients had a ß2m >UNL.

### Histological features and prognosis

**Figure 2:**
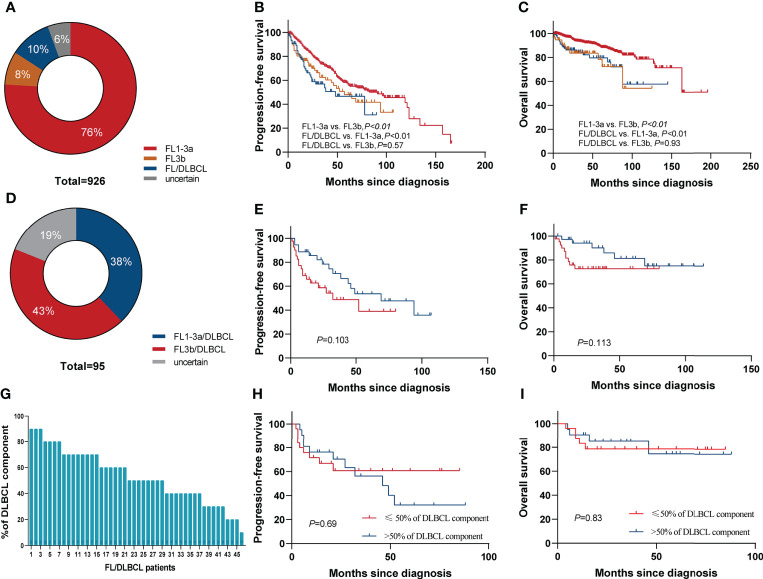
Survival based on histological grade of FL. **(A)** Pie chart showing the proportion of different histological grades of FL. Kaplan–Meier curves of **(B)** PFS and **(C)** OS for FL grade 1–3a, grade 3b, and FL/DLBCL groups. **(D)** Pie chart showing the proportion of FL1–3a/DLBCL and FL3b/DLBCL. Kaplan–Meier curves of **(E)** PFS and **(F)** OS for FL1–3a/DLBCL and FL3b/DLBCL. **(G)** The amount of the DLBCL component ranged from 5% to 90%. **(H)** PFS and **(I)** OS according to the percentage of DLBCL component in FL/DLBCL patients.

In total, 926 patients were diagnosed with FL [grade 1–3a, 703 cases (76%); grade 3b, 76 (8%); FL/DLBCL, 95 (10%); uncertain grades, 52 (6%)] (**Figure 2A**). The 5-year PFS rates of grade1–3a, grade 3b, and FL/DLBCL were 57.8%,46.7%, and 44.8%, respectively, the 5-year OS rates were 88.7%, 78.3%, and 76.1%, respectively. Interestingly, we found that there was no significant difference in 5-year PFS (*p*=0.536) and 5-year OS rates (*p*=0.932) between grade 3b group and FL/DLBCL group (**Figures 2B, C**). To further analyze the impact of different FL grades on FL/DLBCL (**Figure 2D**), we analyzed the prognosis of FL 1–3a/DLBCL and FL 3b/DLBCL. The results showed that the difference was not statistically significant (**Figures 2E, F**). No significant differences were seen between FL 1/2 and FL 3a in terms of PFS, whereas FL 1/2 patients had a longer OS than FL 3a (*p*=0.03) and FL3b (*p*<0.01) (**Supplementary Figure S1**). In addition, the amount of the DLBCL component in FL/DLBCL cases ranged from 5% to 90% (**Figure 2G**). Among FL/DLBCL cases, 31 (33%) were of GCB origin, 17 (18%) ABC, and 47(49%) unclassified. PFS and OS curves according to the amount and cell of origin of DLBCL component are depicted in **Figures 2H, I** and **Supplementary Figure S2**, showing no differences.

### Treatment

#### Overview of treatment regimens and clinical responses

Among 703 patients with grade1–3a FL, 10.2% (n=72) of patients were administered with WW; a total of 631 patients received systemic chemotherapies. The most common treatment regimens were rituximab, cyclophosphamide, doxorubicin, vincristine, and prednisone (RCHOP)-like chemotherapy in 60.9% (n=428). The overall response rate (ORR; CR plus PR) to initial therapy among all treated FL patients was 78.3%, with 39% (n=246) achieving complete response (CR). The treatment modalities and responses of different groups are shown in **Table 2**.

**Table 2:** The first-line treatment model and clinical response in the 1–3a FL cohort.

Treatment	Patients,n	ORR%	CR, n (%)	PR, n (%)	SD, n (%)	PD, n (%)	Censored, n(%)
RCHOP-like	428	81.1	187(43.7%)	160(37.4%)	9(2.1%)	11(2.6%)	61(14.3%)
CHOP-like	127	72.4	33(26%)	59(46.4%)	6(4.7%)	5(3.9%)	24(18.9%)
RFlu-based	23	69.6	8(34.8%)	8(34.8%)	1(4.3%)	1(4.3%)	5(21.7%)
Flu-based	31	80.6	11(35.5%)	14(45.2%)	1(3.2%)	0	5(16.1%)
Chemo-free	18	77.8	7(38.9%)	7(38.9%)	2(11.1%)	0	2(11.1%)
Radiotherapy	4	75	1(25%)	2(50%)	0	1(37.4%)	0
Watch and wait	72	/	/	/	/	/	/
Total	703	78.3	247(39%)	250(39.6%)	19(3%)	18(2.9%)	97(13.8%)

ORR, objective response rate; CR, complete response; PR, partial response; SD, stable disease; PD, progressive disease; Flu, fludarabine; chemo-free, R or R^2^.

#### Comparison of survival between distinct clinical responses

**Figure 3:**
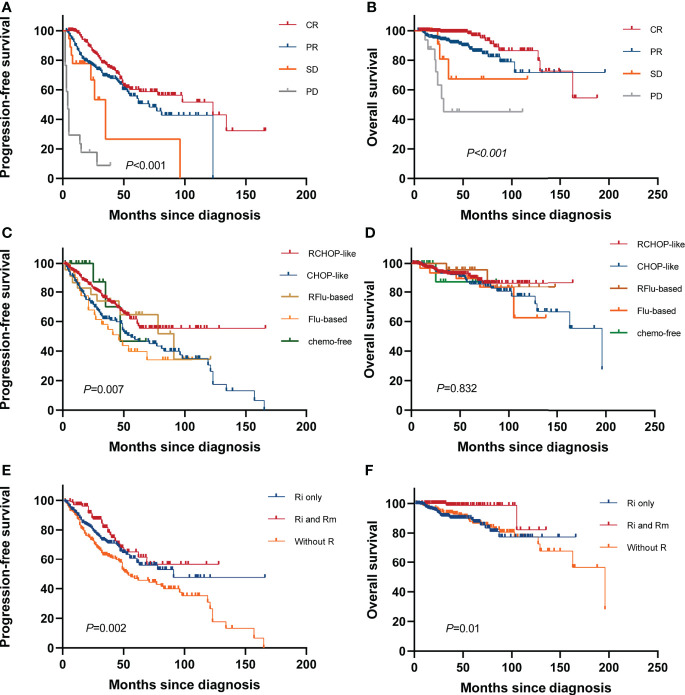
Survival based on treatment. Kaplan–Meier curves of **(A)** PFS and **(B)** OS based on remission status at the end of first-line therapy. **(C)** PFS and **(D)** OS for all systemic therapy regimens. **(E)** PFS and **(F)** OS according to different rituximab administrations [no rituximab treatments (without R), first-line induction chemotherapies with rituximab (Ri only), followed by rituximab maintenance (Ri and Rm)].

A total of 534 patients were evaluated for response; 97 patients were excluded due to the following: two patients progressed or died before any response assessment and were considered non-responders, 10 patients were being treated and had not yet been evaluated for efficacy, and 85 patients could not be evaluated due to lack of imaging data. The 3-year OS rate of CR, PR, SD, and PD groups were 97.8%, 92.4%, 80.8%, and 45.1%, respectively (*p*<0.001), and the 3-year PFS rate were 76.7%, 69.3%, 26.7%, and 8.8%, respectively (*p*<0.001). Achievement of CR was strongly associated with longer survival, which was independent of chemotherapy regimen (**Figures 3A, B**).

#### Comparison of survival between distinct treatment regimens

At the end of induction therapy, the five arms (RCHOP-like, CHOP-like, RFlu-based, Flu-based, and chemo-free group) had similar ORR: 81.1%, 72.4%, 69.6%, 80.6%, and 77.8%, respectively (*p*=0.227). The difference in CR rates between the five arms was statistically significant: 43.7%, 26%, 34.8%, 35.5%, and 38.9%, respectively(*p*=0.01). The CRR among different therapeutic groups is shown in **Supplementary Figure S3**. The estimated 5-year PFS were 61.6%, 47.9%, 64.7%, 39.7%, and 46.7%, respectively (*p*=0.007); the 5-year OS were 91.4%, 86%, 95.7%, 89.8%, and 90.7%, respectively (*p*=0.832). Kaplan–Meier curves of all chemotherapy regimens are shown in **Figures 3C, D**. Based on these results and the proven value of rituximab in FL, we further analyzed the hematological toxicity of RCHOP-like, RFlu-based, and chemo-free groups. Study regimens were generally well tolerated. Patients treated with the three regimens are less likely to have grade 3–4 anemia, thrombocytopenia, neutropenia, and leukopenia. There is no significant difference in hematological toxicity among the three treatment regimens (**Supplementary Table S2**). Additionally, we also analyzed the outcomes of stage I–II patients; there is no statistically significant difference between the watch-and-wait group and the systemic treatment group (**Supplementary Figure S4**).

#### The value of rituximab

Information on the use of rituximab for induction and maintenance was available for 631 patients. In these patients, the 5-year PFS and OS were 60.7% (95% CI, 56.6–64.8) and 88.6% (95% CI, 86.2–91.0) for patients who received rituximab in induction only (Ri), and 62.5% (56.2–68.8) and 98.6% (97.3–99.9) for those who received it for both induction and maintenance (Ri and Rm). Compared with patients not exposed to rituximab (without R), “Ri and Rm” was associated with increased PFS with a hazard ratio (HR) of 0.50 (95% CI, 0.32–0.78; *p*=0.001) and OS with an HR of 0.17 (95% CI, 0.04–0.75; *p*=0.003) (**Figures 3E, F**).

#### Incidence and outcomes of patients with HT

**Figure 4:**
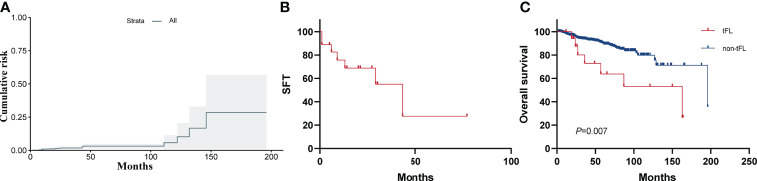
**(A)** Cumulative incidence of histological transformation (CI-HT) as a first event in patients with FL. The shaded area shows the 95% CI. The CI-HT at 5 years was 4.7%. **(B)** Survival following histological transformation (SFT). Overall 3-year SFT was 55%. **(C)** OS for patients with or without histological transformation (HT).

By the follow-up date, 18 patients eventually developed biopsy-proven HT. Ki-67 ranges from 5% to 50% (median, 30%) and 40% to 90% (median, 60%) at initial diagnosis and transformation, respectively. This suggests that the proliferation index increases during transformation. The lymphoma subtype at the time of transformation was DLBCL in 13 patients and high-grade B-cell lymphoma not otherwise specified in 5 patients (of which 1 patient was with MYC and BCL2 rearrangements, considering a double-hit lymphoma). For the latter, immunohistochemistry was performed to stain the markers of TDT to exclude lymphoblastic lymphoma/leukemia; all were negative for TDT. None of the FL patients transdifferentiated into histiocytic sarcoma. Considering the 18 patients who developed HT, median time to transformation was 23.5 months after diagnosis (range, 2–146 months). The CI-HT at 5 years was 4.7% (95% CI, 3.5–5.9) (**Figure 4A**). After a median follow-up after HT of 13.5 months (range, 1–77 months), seven patients had died, with a 3-year SFT of 55% (95% CI, 40–70, **Figure 4B**). The 5-year OS rate from diagnosis was significantly worse for patients with tFL than for patients without transformation (63.7%; 95% CI, 50.4–77.0 *vs*. 89.7%, 95% CI, 88.1–91.3; *p*=0.007) (**Figure 4C**).

#### POD24

**Figure 5:**
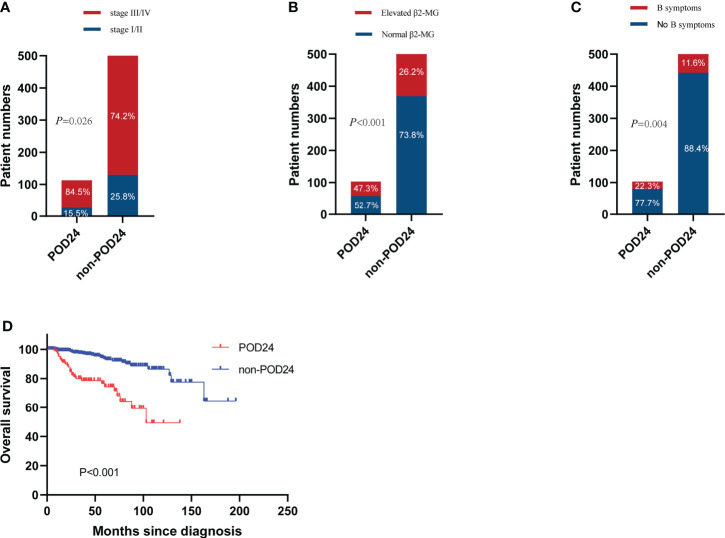
Incidence of POD24 in three states. **(A)** Stage III–IV, **(B)** elevated β2-MG, and **(C)** B symptoms. **(D)** OS for patients with or without POD24.

A total of 631 patients with grade 1–3a FL were treated with first-line treatment. After the exclusion of 5 patients who died without progression within 24 months from treatment start and 23 patients lost to follow-up within 24 months without progression, we included 603 evaluable patients in this analysis. Of these patients, 103 (17.1%) had POD24. The clinical characteristics of the POD24 and non-POD24 subgroups are summarized in **Table 3**. Patients in the POD24 group were more likely to have advanced stage than the non-POD 24 subgroups: stage III–IV (84.5% *vs*. 74.2%, p=0.026), elevated β2-MG (43.7% *vs*. 26.2%, *p*<0.001), B symptoms (22.3% *vs*. 11.6%, p=0.004), and the difference were statistically significant (**Figures 5A–C**).

**Table 3:** Baseline demographics and clinical characteristics of POD24 and non-POD24 group.

Characteristic	POD24	Non-POD24	*p-*value (χ^2^)
	n (%)	n (%)	
No. of patients	103	500	
Age (years), median(range)	51(28–79)	54(24–82)	
Age>60 years	32(31.1%)	146(29.2%)	0.705
Male gender	55(53.4%)	233(46.6%)	0.208
Stage III/IV	87(84.5%)	371(74.2%)	0.026
Hb<120g/L	23(22.3%)	87(17.4%)	0.238
Elevated β2-MG	45(43.7%)	131(26.2%)	<0.001
Elevated LDH	26(25.2%)	92(18.4%)	0.111
B symptoms	23(22.3%)	58(11.6%)	0.004

With a median follow-up of 38 months (range, 1–196 months), the median survival time of the POD24 group was 126 months (95% CI, 85–167 months) compared to the non-POD24 group not reached. Among patients who experienced POD24, the 5- and 10-year OS rates were 74.6% and 51.7%, respectively, and in the non-POD24 group, the rates were 94.6% and 87.4%, respectively (HR, 0.21; 95% CI, 0.12–0.36, *p*<0.001). This is obvious from **Figure 5D**, where the main difference between the two groups was the late survival rate.

#### Comparison of the FLIPI, FLIPI2, and PRIMA-PI

**Figure 6:**
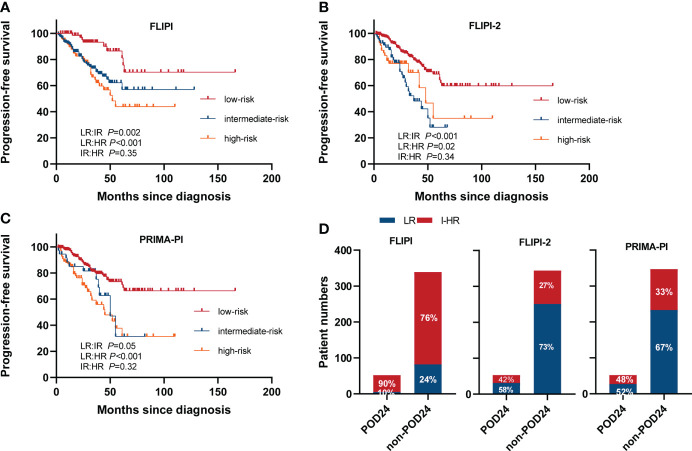
PFS in different scoring systems. **(A)** FLIPI, **(B)** FLIPI2, and **(C)** PRIMA-PI. **(D)** Accuracy of the three risk models to predict POD24 status. LR, low risk; IR, intermediate risk; HR, high risk.

We compared three clinical prognostic models (the FLIPI, the FLIPI2, and PRIMA-PI) to identify the optimal model for Chinese FL patients undergoing chemoimmunotherapy. PFS curves according to the score are presented in **Figures 6A–C**. All three models significantly identified PFS between low risk (LR) and intermediate risk (IR), and LR and high risk (HR). However, they could not effectively distinguish PFS between IR and HR subgroups (*p*>0.05 for all). Hence, we divided patients into two subgroups of LR and intermediate–high risk (I-HR) to further analyze the predictive potency of these models on POD24. Sensitivity for I-HR score to predict POD24 in the cohort was 90% with FLIPI compared with 42% for FLIPI-2 and 48% for PRIMA-PI. Specificity for POD24 was 24% with FLIPI compared with 73% for FLIPI-2 and 67% for PRIMA-PI **(** **Figure 6D**). The FLIPI-2 thus showed the highest specificity, reflecting the prediction of correctly classifying as LR patients, but the FLIPI had the highest sensitivity to predict the risk of progression at the critical. OS curves according to the FLIPI, the FLIPI2, and PRIMA-PI score are presented in **Supplementary Figures S5A–C**. PRIMA-PI can significantly distinguish the OS between LR and HR, and IR and HR, while FLIPI and FLIPI2 cannot identify OS between IR and HR. Therefore, we cautiously draw the following conclusions: PRIMA-PI can better identify HR patients when predicting OS, while FLIPI tends to predict PFS.

## Discussion

FL is a common B-cell lymphoma and is characterized by its high heterogeneity in clinical characteristics and outcomes (5). A previous analysis by our center of a single-disease database of more than 3,000 lymphomas found that FL accounted for 24.13% of NHL, similar to that reported earlier for Western FL patients (22%–35%) (19) but slightly higher than that reported in Chinese cohorts (8.1%–23.5%) (20). The median age of onset was 54 years old in our cohorts, similar to that reported earlier for Chinese FL patients (49–53 years) (21, 22) but much younger than that reported in Western cohorts (60–65 years) (7, 23). Several studies from China showed the BMI (bone-marrow infiltration) rate of 15.2%–28% in patients with FL, similar to our results, significantly lower than that in Western patients (40%–70%) (5, 13). Approximately 90.9% of Chinese FL patients have ECOG ≤ 1, which is consistent with 91% previously reported in Western countries (24). Other clinical characteristics of FL patients in our cohort are comparable to those reported in FL patients in China and the Western cohort (19, 21–25).

Some patients with FL showed related DLBCL components in lymph node biopsy at the time of diagnosis, and the biological and clinical significance remain unclear (10–12). One of the main results of this article is that patients with FL/DLBCL had a poorer outcome than those with FL pure grade 1–3a but similar to grade 3b. The PFS and OS of FL 1–3a/DLBCL is similar to that of FL 3b/DLBCL. In a different setting, FL1-3a/DLBCL seems to have a better OS than FL 3b/DLBCL (16). Concomitant DLBCL was more common in FL 3b (42.3% of FL 3b showed DLBCL component) than in FL 1–3a (37.9%), in concordance to previous reports (16, 26). In the present study, FL 1/2 showed no significant difference in PFS compared with FL 3a but showed a better OS compared with FL3a and FL 3b among all the patients. Although OS is the gold standard for evaluating the efficacy of cancer treatment, its utility in relatively indolent diseases is challenged. In FL, PFS is a well-established parameter for evaluating treatment outcome. This result suggested that FL 1/2 was closely related to FL3a as reflected by a similar indolent clinical course.

Patients with high tumor burden disease meeting GELF or BNLI criteria are typically symptomatic and require treatment with rituximab for better survival benefit (27). Data on rituximab were available for all patients, out of which 489 (69.6%) patients received this agent at least once during the induction or maintenance phase. The use of rituximab increased significantly between the period 2002–2010 and 2011–2020 from 32.2% to 69.5% (*p <*0.001). Compared with chemotherapy alone, immunochemotherapy has been associated with higher response, PFS, and OS rates (28, 29). The PRIMA study long-term follow-up demonstrates that R-maintenance after induction immunochemotherapy provides a significant long-term PFS benefit over observation (mPFS, 10.49 years *vs*. 4.06 years, HR=0.61, *p*<0.0001). Furthermore, more than half of the patients in the R group remain free of disease progression and have not required new anti-lymphoma treatment beyond 10 years (30). However, a study based on real-world data suggests that the value of rituximab maintenance in FL is limited and, in the absence of clear OS benefits, remains a controversial approach (31). Our study shows that R maintenance after induction immunochemotherapy yielded the most favorable prognosis, which is consistent with the results of another real-world study in Chinese population (21). However, the study did not include bendamustine (B) in combination with rituximab or Obinutuzumab (G)-based chemotherapy, as B and G were not available in China until 2019 (32). Unlike approximately 20% of FL patients treated with observation in the cohorts of Western countries, only 7% of Chinese FL patients were administered with watch and wait (21). In the study, the ORR was 78.3%, and the 5-year PFS and OS of all patients were 57.8% and 88.7%, respectively. Both were similar to those previously observed in Chinese FL patients and several Western cohorts (21, 33, 34). Failure to achieve CR with frontline therapy was also significantly associated with poorer outcomes, suggesting that with the extension of follow-up time, differences in OS among the groups may become apparent. The results of the FOLL05 randomized study clearly demonstrated that R-CHOP chemotherapy regimens lead to significantly improved PFS in patients with previously untreated FL and had a better risk–benefit ratio compared with RFlu-based regimen (34). Therefore, we cautiously conclude as follows: this research cohort seems to support R-CHOP-like followed by R maintenance as a reference plan for the initial management of Chinese FL patients who require active treatment, which was analogous to those observed previously in Western cohorts (30)

Histological transformation to a more aggressive form remains a critical event in the natural history of FL. The CI-HT at 5 years was 4.7% in our study. In a large study analyzing tFL, where HT was also a mandatory criterion, the CI-HT at 5 years was 5% (35), consistent with our results, but lower than previously reported 10%–22% (36). The difference may be due to different criteria applied to patient recruitment and the definition of transformation. In the study, we have analyzed rates and outcomes in a homogeneous cohort of patients with the certainty of having a histologically documented transformation. However, some patients who were clinically suspected of transformation (sudden increase in LDH, rapid discordant localized nodal growth, new involvement of unusual extra-nodal sites, new “B” symptoms, or hypercalcemia) were not classified as tFL (35), mainly because there was not enough evidence to consider them equally. Patients with tFL had poor OS, with a 5-year SFT rate of 25%–48%, consistent with our findings (35, 37). However, due to the small number of transformation cases, the potential genetic alterations associated with transformation could not be revealed. Future studies are important to address this unmet need.

In recent years, studies have found that among FL patients receiving first-line therapy, POD24 after diagnosis is a significant adverse prognostic factor (18, 38). POD24 occurred in 17% and 23% of evaluable GLSG and BCCA patients (39), 17.7% in our study. The results of our study showed that the 5-year OS rates of patients with POD24 and those without POD24 were 75.6% and 92.8%, respectively (*p*<0.001), which was consistent with previous studies. It is worth noting that the immune infiltration^LO^ (i.e., low programmed death-ligand 2, PD-L2) subset of patients with FL was enriched for POD24 events. Therefore, assessment of immune infiltration by PD-L2 expression seems to be a promising tool with which to help identify patients who are at risk for POD24 (40).

Various clinical and clinicogenetic scores are available for predicting prognosis in patients with previously untreated FL who are about to commence first-line therapy (13, 41–43). In our study, we compared three clinical prognostic models to identify the optimal model for Chinese FL patients undergoing chemoimmunotherapy. The results showed that currently applied clinical prognostic models such as FLIPI, FLIPI2, and PRIMA-PI have suboptimal effect in predicting the intermediate- and high-risk poor prognosis subgroups. The FLIPI-2 showed the highest specificity of 73% to predict POD24, reflecting the prediction of correctly classifying as LR patients, but the FLIPI-1 had the highest sensitivity to predict the risk of progression at the critical.

In recent years, even if FL patients were found to be in the early stages (stage I or II), doctors will incorporate rituximab into the treatment strategy of FL patients to prolong survival, rather than the so-called “watch and wait” strategy. Active therapies, mainly including rituximab, tend to start in the earlier stages in Japan (44). Our study shows that for stage I–II patients, watchful waiting can also achieve good outcomes if the patient is not motivated to treat and has no indication for treatment.

In summary, this large retrospective study revealed the clinical features and outcomes of Chinese patients with FL in the real world; the majority had advanced-stage disease and ECOG ≤ 1 but less frequently infiltrated bone marrow. RCHOP-like followed by R maintenance was a valid regimen for Chinese FL patients. Patients with stage III–IV, elevated β2-MG, and B symptoms seem to be more prone to POD24.The study provided novel data on prognostic factors and primary treatment of FL, applicable to routine clinical practice.

## Data availability statement

The original contributions presented in the study are included in the article/**Supplementary Material**. Further inquiries can be directed to the corresponding authors.

## Ethics statement

The study was reviewed and approved by the Research Ethics Committee of the Tianjin Medical University Cancer Institute and Hospital. Written informed consent for participation was not required for this study in accordance with the national legislation and the institutional requirements.

## Author contributions

HZ and XW contributed to study design; FG, TZ, XiaL and ZQu acquired the data and performed the analysis; XianL, LL, LQ, ZQi and SZ collected the clinical information; WG and BM checked the pathology; XR supported good suggestions; FG wrote the draft; XW revised the manuscript. All authors contributed to the article and approved the submitted version.

## Funding

This study was supported by the Scientific Research Project of Tianjin Educational Committee (grant no. 2019KJ191).

## Conflict of interest

The authors declare that the research was conducted in the absence of any commercial or financial relationships that could be construed as a potential conflict of interest.

## Publisher’s note

All claims expressed in this article are solely those of the authors and do not necessarily represent those of their affiliated organizations, or those of the publisher, the editors and the reviewers. Any product that may be evaluated in this article, or claim that may be made by its manufacturer, is not guaranteed or endorsed by the publisher.
