# Supplementary material for: Clinical features and outcomes of patients with follicular lymphoma: A real-world study of 926 patients in China
Source: Front Oncol. 2022 Sep 16;12:863021. doi: 10.3389/fonc.2022.863021 (PMC9522898; doi:10.3389/fonc.2022.863021)
Supplement: Supplementary file 1 [file DataSheet_2.docx]

**Supplemental Figure legends:**


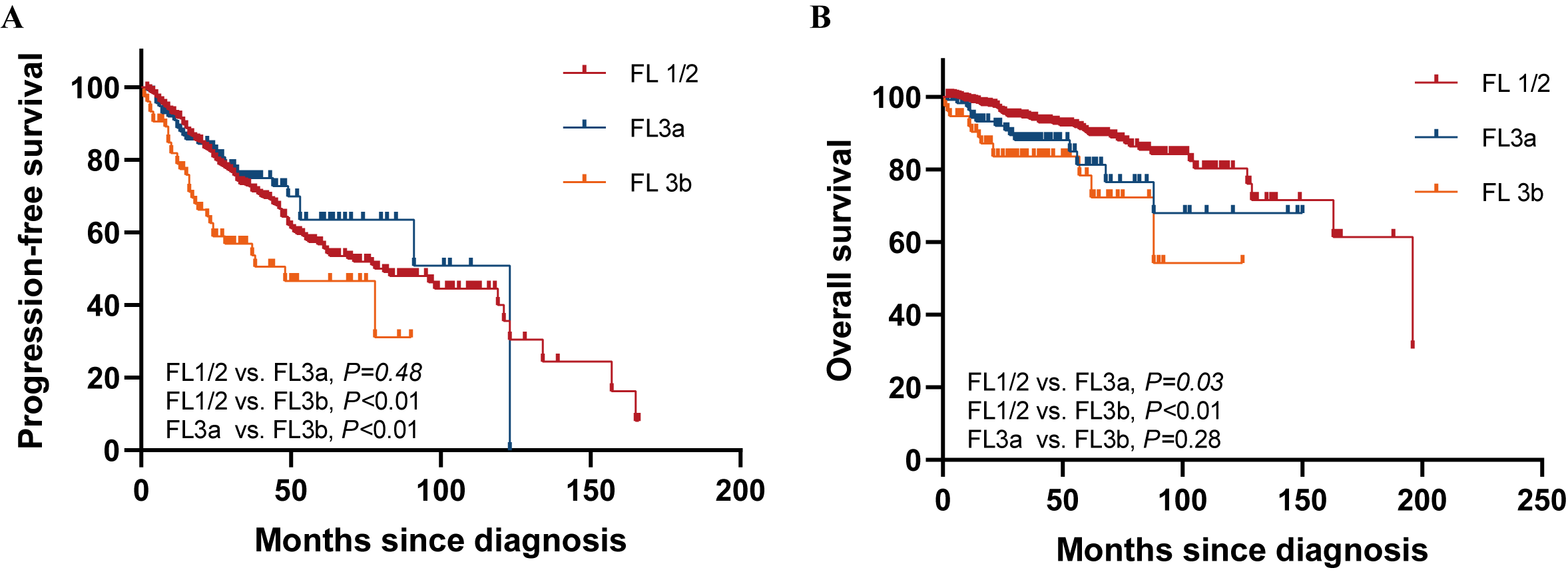


**Figure S1:**(**A**) PFS and (**B**) OS for FL1/2, FL3a and FL 3b groups.

**
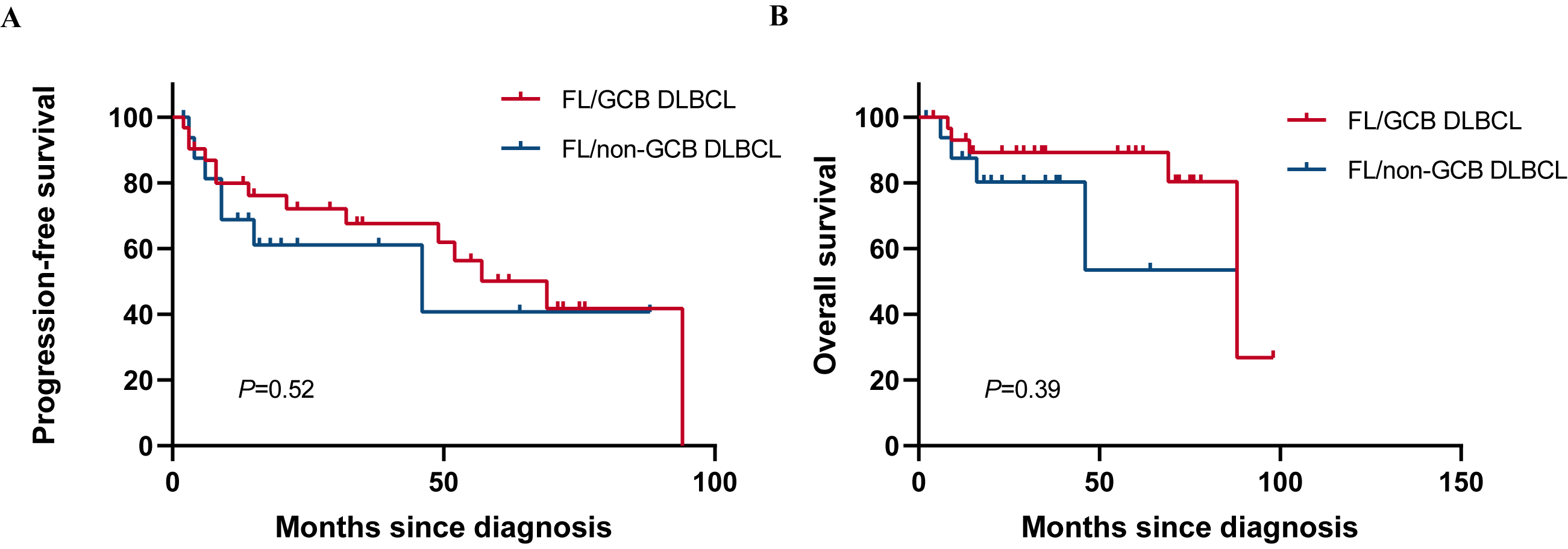
**

**Figure S2:**(**A**) PFS and (**B**) OS according to the cell of origin in FL/DLBCL patients.

**
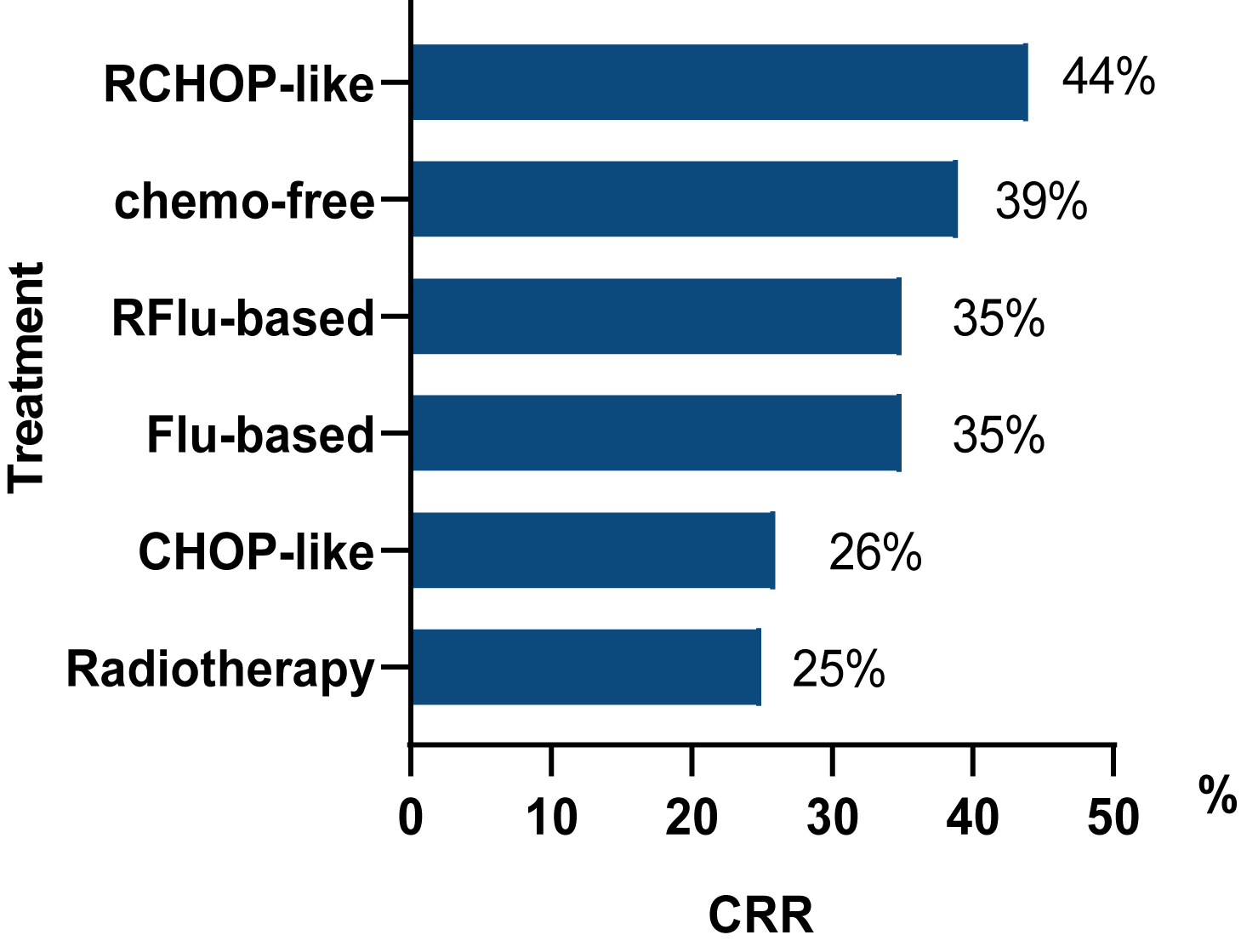
**

**Figure S3.** The CRR among different therapeutic groups.


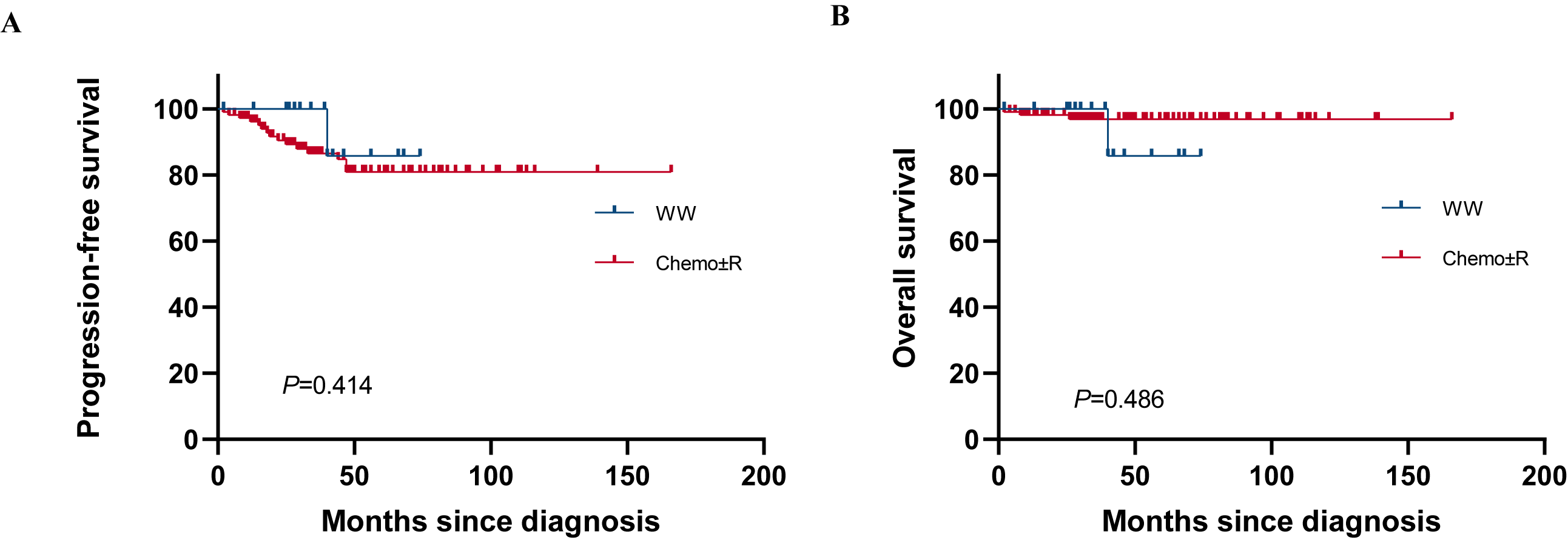


**Figure S4.** For stage I-II patients, Kaplan-Meier curves of (**A**) PFS and (**B**) OS for WW and chemo±R.


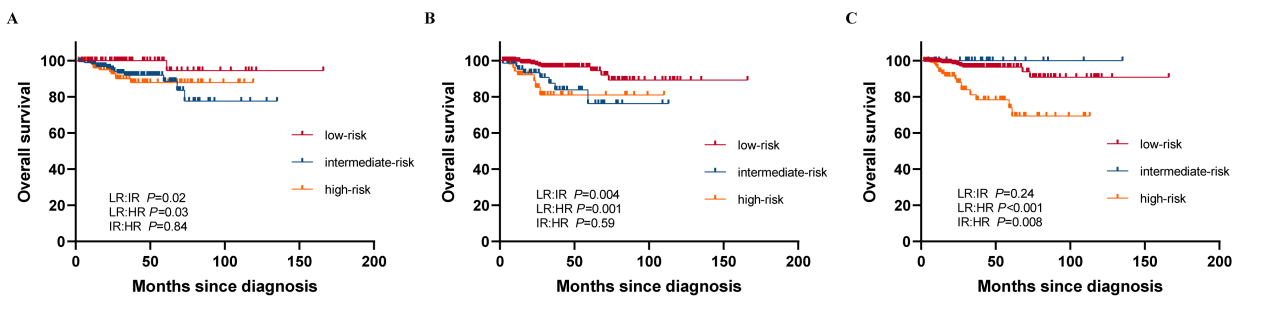


**Figure S5.** OS in different scoring systems.(A) FLIPI.(B) FLIPI2. (C) PRIMA-PI.
