# Supplementary material for: Clinical features and outcomes of patients with follicular lymphoma: A real-world study of 926 patients in China
Source: Front Oncol. 2022 Sep 16;12:863021. doi: 10.3389/fonc.2022.863021 (PMC9522898; doi:10.3389/fonc.2022.863021)
Supplement: Supplementary file 2 [file DataSheet_1.doc]

| **Table S1**. The criteria for assessment of tumor burden in FL | | | | |
| --- | --- | --- | --- | --- |
| GELF criteria | | |  | BNLI criteria |
| Involvement of 3 or more nodal sites measuring 3 cm or greater | | |  | Presence of pruritus or B symptoms |
|  |
| Nodal or extra nodal tumor mass 7 cm or greater | | |  | Rapid disease progression in preceding 3 months |
|  |
| Presence of B symptoms |  |  |  | Life threatening organ involvement |
| Splenomegaly | | |  | Bone marrow infiltration causing cytopenias |
|  |
| Presence of ascites or pleural effusions | | |  | Localized bone lesions |
|  |
| Presence of circulating malignant cells (leukaemic phase) | | |  | Renal infiltration |
|  |
| Presence of cytopenias | | |  | Macroscopic liver involvement |
| This table comes from Carla Casulo | | | | |

| **Table S2**. Hematologic toxicity by study groups | | | | | | | |
| --- | --- | --- | --- | --- | --- | --- | --- |
|  | RCHOP-like (n=428; %) | | RFlu-based (n=23; %) | | chemo-free (n=18; %) | |  |
| Toxicity (grade) | 1-2 | 3-4 | 1-2 | 3-4 | 1-2 | 3-4 | *P* value * |
| Anemia | 2 | 0 | 1 | 0 | 0 | 0 | 0.071 |
| Neutropenia | 36 | 5 | 2 | 0 | 4 | 0 | 0.225 |
| Thrombocytopenia | 17 | 0 | 0 | 0 | 0 | 0 | 0.43 |
| leukopenia | 48 | 2 | 2 | 0 | 2 | 0 | 0.905 |
| NOTE. Toxicity data were available for 465 of 469 patients. | | | | | |  |  |
| * χ^2^ or Fisher's exact test | | |  |  |  |  |  |
